# A Self-Powered Breath Analyzer Based on PANI/PVDF Piezo-Gas-Sensing Arrays for Potential Diagnostics Application

**DOI:** 10.1007/s40820-018-0228-y

**Published:** 2018-11-12

**Authors:** Yongming Fu, Haoxuan He, Tianming Zhao, Yitong Dai, Wuxiao Han, Jie Ma, Lili Xing, Yan Zhang, Xinyu Xue

**Affiliations:** 1 School of Physics, University of Electronic Science and Technology of China, Chengdu, 610054 People’s Republic of China; 2 College of Physics and Electronics Engineering, Shanxi University, Taiyuan, 030006 People’s Republic of China; 3 College of Sciences, Northeastern University, Shenyang, 110004 People’s Republic of China

**Keywords:** Polyaniline, Polyvinylidene fluoride, Piezoelectric, Sensor array, Diagnostics, Breath analyzer

## Abstract

The increasing morbidity of internal diseases poses serious threats to human health and quality of life. Exhaled breath analysis is a noninvasive and convenient diagnostic method to improve the cure rate of patients. In this study, a self-powered breath analyzer based on polyaniline/polyvinylidene fluoride (PANI/PVDF) piezo-gas-sensing arrays has been developed for potential detection of several internal diseases. The device works by converting exhaled breath energy into piezoelectric gas-sensing signals without any external power sources. The five sensing units in the device have different sensitivities to various gas markers with concentrations ranging from 0 to 600 ppm. The working principle can be attributed to the coupling of the in-pipe gas-flow-induced piezoelectric effect of PVDF and gas-sensing properties of PANI electrodes. In addition, the device demonstrates its use as an ethanol analyzer to roughly mimic fatty liver diagnosis. This new approach can be applied to fabricating new exhaled breath analyzers and promoting the development of self-powered systems.

**Electronic supplementary material:**

The online version of this article (10.1007/s40820-018-0228-y) contains supplementary material, which is available to authorized users.

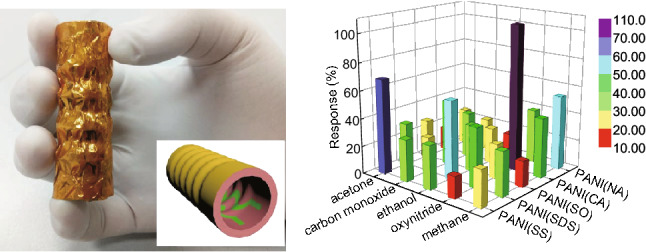

## Highlights

- A self-powered breath analyzer based on polyaniline/polyvinylidene fluoride (PANI/PVDF) piezo-gas-sensing arrays was developed for a potential diagnostics application.
- The device works by converting energy from exhaled breath into electrical sensing signals without any external power sources.
- The working principle can be attributed to the coupling of in-pipe gas-flow-induced piezoelectric effect of PVDF bellows and gas-sensing effect of PANI electrodes.

## Introduction

In recent years the increasing morbidity of internal diseases induced by unwholesome diet and working behaviors has posed a serious threat to human health and quality of life [1–3]. Early detection and treatment play key roles in improving the cure rate of the patients [4]. Although conventional blood examination methods have been developed with good sensitivity and stability [5], a noninvasive, portable, and convenient diagnostic method is urgently needed for a wide range of early diagnoses and high-risk population screening [6]. As an important physiological process for human beings, breath is one of the most important ways to exchange substances between the human body and outside world. Various studies suggest that exhaled breath, containing a large number of metabolic products, includes gas species and concentrations closely associated with human health as indicators of certain diseases [7–12]. For example, ethanol in exhaled breath is recognized as a gas marker of fatty liver; oxynitride (NO_*x*_) is a gas marker of airway inflammation; acetone is a gas marker of diabetes; methane (CH_4_) is a gas marker of liver cirrhosis; and carbon monoxide (CO) is a gas marker of asthma. However, exhaled breath analyzers are restricted by possible limitations, such as high cost, structure complexity, need for high-quality materials, and reliance on external power sources.

Self-powered systems aimed at powering portable and wearable electronics with human motion have been developed based on piezoelectric or triboelectric nanogenerators [13–19]. In addition, conducting polymer-based gas sensors are widely used in room-temperature detection of exhaled gas markers [20–23]. Compared with triboelectric nanogenerators [24, 25], piezoelectric nanogenerators can work without the contact–separation process between two materials, which is more suitable for constructing portable self-powered devices. By simultaneously carrying out power generation and gas sensing, the integration of the piezoelectric nanogenerator and gas sensor in a single device may be a feasible way to realize a self-powered exhaled breath analyzer. A novel and distinct device architecture should be developed adapting to the convenience and compatibility of exhaled breath analysis.

In this paper, we report a self-powered breath analyzer based on polyaniline/polyvinylidene fluoride (PANI/PVDF) piezo-gas-sensing arrays for a potential diagnostics application. PANI is an easily synthesized conducting polymer that is widely used in room-temperature gas-sensing applications [23]. PVDF is a piezoelectric polymer with a high piezoelectric coefficient and flexibility [26]. Based on coupling of the in-pipe gas-flow-induced piezoelectric effect of PVDF and gas-sensing properties of PANI electrodes, the exhaled breath analyzer can convert energy from exhaled breath into piezoelectric gas-sensing signals. The device consists of five different sensing units, with each sensing unit having favorable selectivity to a particular gas marker. The sensing signals of every sensing unit hold a proportional relationship with gas concentration in a wide range (from 0 to 600 ppm), along with outstanding room-temperature response/recovery kinetics. This work launches a new working principle in the exhaled breath detection field and greatly advances the applicability of self-powered systems.

## Experimental Procedures

### Fabrication of PANI Electrodes

First, a piece of copper foil (5 cm × 5 cm × 10 μm) was covered with a photoresist pattern by photolithography. Then, the copper foil was wet-etched by soaking with aqueous sodium persulfate (0.5 mol L^−1^) at 50 °C for 2 min, followed by immersion into developer for 30 s to remove the residual photoresist. Second, PANI derivatives were deposited on Cu substrate by electrochemical polymerization. The growing solution contained equal molar concentrations (0.2 mol L^−1^) of dopant and aniline monomer. Sodium sulfate, sodium dodecylbenzene sulfonate, sodium oxalate, camphorsulfonic acid, and nitric acid were used as dopant sources in each of the five PANI derivatives. The Pt wafer, Ag/AgCl electrode, and Pt wire served as the working, reference, and counter electrodes, respectively. The electrochemical reaction was employed at 1.2 V with 0.05 V s^−1^ for 200 s to polymerize PANI. Finally, twist pattern PANI electrodes were obtained by etching in aqueous sodium persulfate (0.5 mol L^−1^) at 50 °C for 2 min to remove the copper substrate.

### Device Fabrication

PVDF gel was obtained by adding 1 g of PVDF powder into 10 mL of acetone and stirring at 60 °C for 30 min. Then, the PVDF gel was spin-coated on PANI electrodes at 200 rpm for 30 s and dried at room temperature for 2 h to form PVDF/PANI film. To enhance the piezoelectricity of PVDF, the film was polarized under an electric field of 20 kV mm^−1^ at 80 °C for 30 min. A 100-nm Cu film was deposited on the back of PVDF by electron beam evaporation. Finally, PANI/PVDF bellows was obtained by extrusion molding at 60 °C for 24 h in a 3D-printed model.

### Characterization and Measurements

For accurate measurement, five PANI electrodes were separately glued to a copper wire as working electrodes, and a copper wire was glued to the back of the Cu film as a shared counter electrode. During the test, the device was placed at one end of a gas pipe connected to a gas cylinder and air compressor. The rate and concentration of gas flow were controlled by gas flowmeters. The gas-sensing performances were studied by measuring the output current in the circuit under different conditions. The gas flow rate was held at 8 m s^−1^ unless specified otherwise. The output current was measured by a low-noise current preamplifier (SR570, Stanford Research Systems) and collected by a data acquisition card (PCI-1712, Advantech) in the computer. The morphology and composition of PANI derivatives were investigated by a scanning electron microscope (SEM; Hitachi S4800) with an energy-dispersive spectrometer (EDS).

## Results and Discussion

**Fig. 1:**
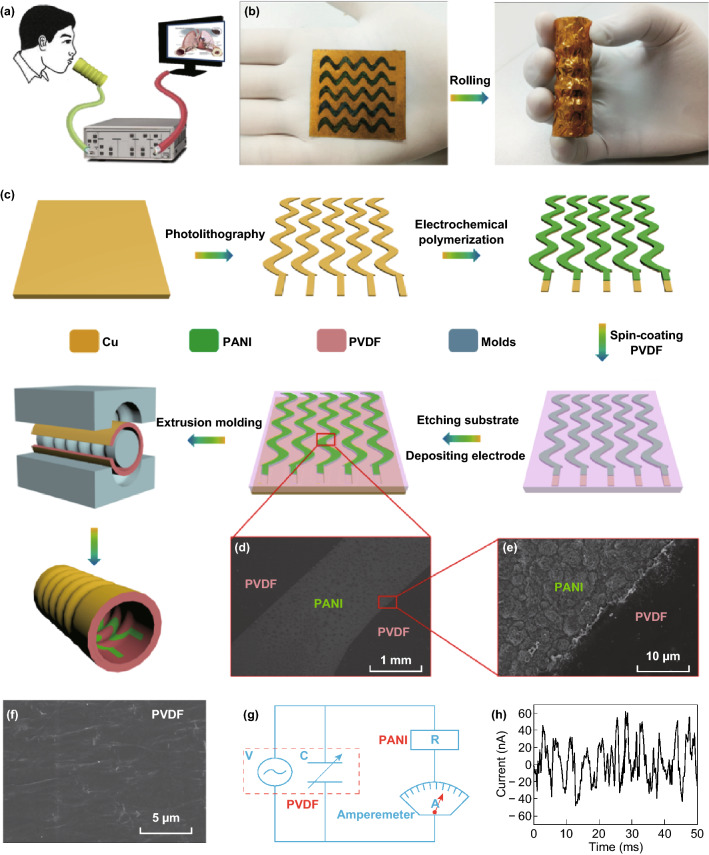
Structure and fabrication of the device. **a** Proposed concept. **b** Photographs of un-rolled PANI/PVDF film and as-fabricated bellows. **c** Fabrication process of the device. **d** SEM image of PANI/PVDF. **e** High-magnification SEM image of PANI/PVDF interface. **f** SEM image of PVDF film. **g** Lumped parameter equivalent circuit model of the device. **h** A few cycles of the output current

Figure 1a is a schematic diagram of the proposed working mechanism of a self-powered exhaled breath analyzer. As a human subject blows into the analyzer, the current signal generated by PANI/PVDF piezoelectric bellows can be measured through a current amplifier and displayed on a computer. Amplitude of the current signal is related to the gases detected in exhaled breath, such that the characteristic of exhaled breath is deduced by calculating the variation of measured signals. The photograph of un-rolled PANI/PVDF film and PANI/PVDF bellows is shown in Fig. 1b. The basic structure of the device mainly consists of three functional parts: Twist PANI patterns function as both working electrodes and gas-sensing material; PVDF film works as the power source through the in-pipe gas-flow-induced piezoelectric effect; and Cu film on the back works as the shared counter electrode. As the bellows configuration was widely used in mechanical energy conversion by extending/retracting along the radial direction [27, 28], the PVDF film is formed into bellows to productively enhance the efficiency of converting breath energy into piezoelectricity. In the as-fabricated device, the thickness of PVDF and PANI is uniform and the 100-nm Cu film is deposited on the whole back surface of PVDF as the shared counter electrode. There are five individual PANI electrodes in one as-fabricated device, and each electrode can be separately connected with an external circuit for electrical measurements, as shown in Fig. S1. The twist pattern structure of PANI electrodes is employed to improve the lifetime and stability as the bellows vibrates with high frequency under blowing. The detailed fabrication progress of the device is shown in Fig. 1c. In brief, a piece of Cu foil is etched to a twist pattern by photolithography; five PANI derivatives are separately deposited on twisted Cu electrodes by electrochemical polymerization; PANI/PVDF film is obtained by spin-coating the PVDF gel on PANI electrodes; PANI/PVDF bellows is shaped up from PANI/PVDF film through extrusion forming. Figure 1d shows a typical SEM image of selected region in one sensing unit (nitric acid-doped PANI), demonstrating that the width of PANI electrode is ~ 2 mm. Figure 1e is a SEM image of the PANI/PVDF interface enlarged from Fig. 1d, showing that the height of PANI is lower than that of PVDF. Figure 1f is a SEM image of the PVDF film, demonstrating that the PVDF surface is smooth, and no pores or fractures can be observed.

To propose the working principle, a lumped parameter equivalent circuit model of the self-powered exhaled breath analyzer can be derived from three circuit elements in a circuit (Fig. 1g) [29–33]: The first one is the voltage term, which originates from the generated piezoelectric polar dipoles in PVDF and can be represented by an ideal voltage source (*V*); the second one is a capacitance term, which originates from the inherent capacitance of PVDF between the two electrodes and can be represented by a capacitor (*C*); the third one is a resistance term, which originates from the variation of PANI derivatives influenced by the atmosphere and can be represented by a resistance (*R*). The capacitor and voltage source of PVDF are parallel-connected to each other and series-connected with the resistance of PANI. Figure 1h shows the generated output current in a few cycles, confirming that the output current is an AC electrical signal.

**Fig. 2:**
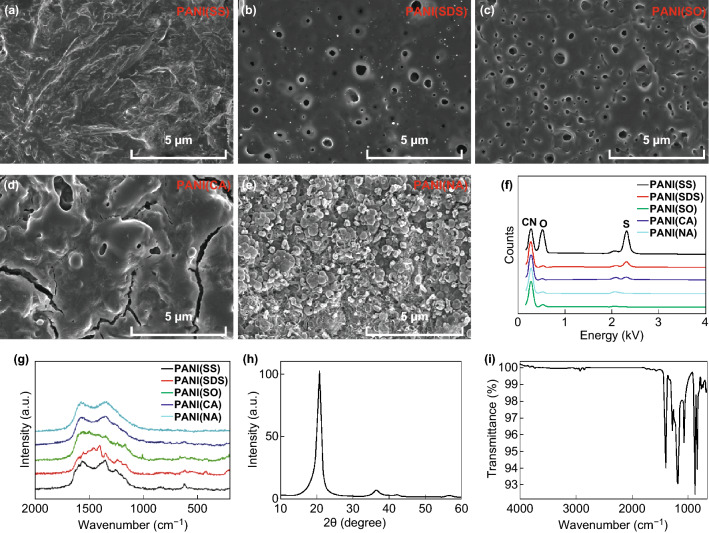
Characterization of the PANI/PVDF. **a**–**e** SEM images of PANI(SS), PANI(SDS), PANI(SO), PANI(CA), and PANI(NA), respectively. **f** EDS spectra of the five PANI derivatives. **g** Raman spectra of the five PANI derivatives. **h** XRD pattern of PVDF film. **i** FTIR spectrum of PVDF film

One can observe that there are five twist PANI electrodes in a single device, and each PANI derivative is doped with different dopant sources. The sensing units are labeled as PANI(SS), PANI(SDS), PANI(SO), PANI(CA), and PANI(NA) with respect to the dopants (sodium sulfate, sodium dodecylbenzene sulfonate, sodium oxalate, camphorsulfonic acid, and nitric acid, respectively). Figure 2a–e shows the SEM images of PANI(SS), PANI(SDS), PANI(SO), PANI(CA), and PANI(NA), respectively, demonstrating the distinctness of the surface morphology of each sample. Figure 2f shows the EDS spectra of the five PANI derivatives. C, N, and O elements can be clearly found in the five samples, while the S element can only be found in PANI(SS), PANI(SDS), and PANI(CA) samples. The absence of the Na element verifies that acid group anions are grafted with PANI chains as a dopant source during the polymerization process [23]. Figure 2g shows Raman spectra of the five PANI derivatives. As observed, the peaks around 1580 cm^−1^ can be assigned to the C=C stretching vibrations of quinoid ring; the peaks around 1500 cm^−1^ can be assigned to the C=C stretching vibrations of benzene ring; and the band around 1350 cm^−1^ provides the information on C~N^+·^ vibrations of delocalized polaronic structures [34]. These results indicate that diverse PANI derivatives are successfully synthesized through electrochemical polymerization and that molecular conformation of PANI can be affected by changing dopants. *I*–*V* curves of the five PANI derivatives are shown in Fig. S2, indicating that the conductivity is significantly influenced by the dopant through charge redistribution in PANI chains [35, 36]. Figure 2h illustrates the XRD pattern of the PVDF film. The dominating peak around 20.72° is narrow and strong, which belongs to (110) and (200) of beta-phase PVDF [37]. Figure 2i is the FTIR spectrum of the PVDF film. The typical peaks around 1400 and 840 cm^−1^ can be indexed to stretching of beta-phase PVDF; the peaks around 1070 and 760 cm^−1^ can be indexed to stretching of alpha-phase PVDF; and the peak around 880 cm^−1^ can be indexed to stretching of amorphous-phase PVDF [38]. These results indicate that the polarized PVDF film is a mixed phase with beta-phase is predominant phase, making the fabricated PVDF an effective piezoelectric film.

**Fig. 3:**
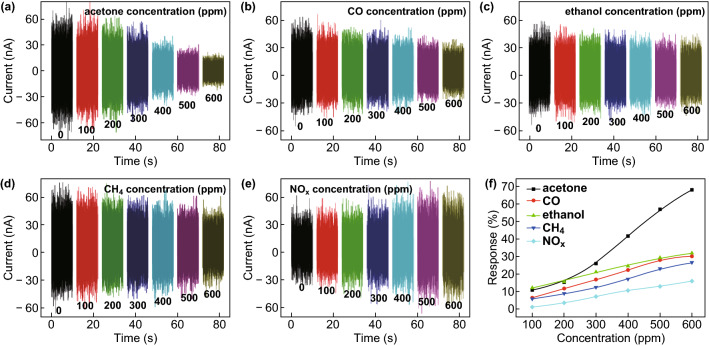
Performances of PANI(SS) sensing unit. **a**–**e** The output current of PANI(SS) in response to acetone, CO, ethanol, CH_4_, and NO_*x*_ with concentrations ranging from 0 to 600 ppm. **f** The relationship between the response and gas concentration

Figure 3 shows the sensing performance of one PANI(SS) sensing unit in different ambient atmospheres with gas concentrations ranging from 0 to 600 ppm. The gas flow rate is held constant at 8 m s^−1^. Figure S3 shows the continuous output current of the PANI(SS) sensing unit under an airflow rate of 8 m s^−1^ for 12 h, exhibiting a slight decrease in the output current after long-time service (the mechanical fatigue of PVDF). Figure 3a–e illustrates that the output current significantly decreases with an increasing concentration of acetone, CO, ethanol, and CH_4_ with discernible differences, but in turn increases with increasing NO_*x*_ concentration. Video S1 shows the real-time sensing process of the PANI(SS) unit against 600 ppm ethanol gas flow. To calculate the relationship between output current and gas concentration, the gas response (*R*_g_) of the device can be simply defined as [39–41]:

1 $$ R_{\text{g}} \% = \frac{{\left| {I_{\text{a}} - I_{\text{g}} } \right|}}{{I_{\text{a}} }} \times 100\% , $$

where *I*_a_ and *I*_g_ represent the output current of the device in air and the output current with a particular concentration of gas marker, respectively. The response curves with respect to acetone, CO, ethanol, CH_4_, and NO_*x*_ are shown in Fig. 3f. With the gas concentration ranging from 100 to 600 ppm in steps of 100 ppm, the response to acetone is 11.0%, 15.1%, 25.7%, 41.8%, 57.0%, and 68.2%; the response to CO is 6.4%, 11.6%, 16.7%, 22.1%, 28.5%, and 30.3%; the response to ethanol is 11.9%, 15.6%, 21.3%, 24.5%, 29.2%, and 31.6%; the response to NO_*x*_ is 1.1%, 3.3%, 7.1%, 10.6%, 12.7%, and 15.8%; and the response to CH_4_ is 5.7%, 8.9%, 12.2%, 17.1%, 23.2%, and 26.4%, respectively. As a maximum, the response to 600 ppm acetone is 68.2%, indicating that the PANI(SS) sensing unit has a promising selectivity against acetone. It can be observed that the gas response is not linear, which may be induced by the nonlinear resistance variation of PANI. Similar nonlinear response curves are often observed in other resistive-type gas sensors, such as metal oxide-based sensors and conducting polymer-based sensors [42, 43].

**Fig. 4:**
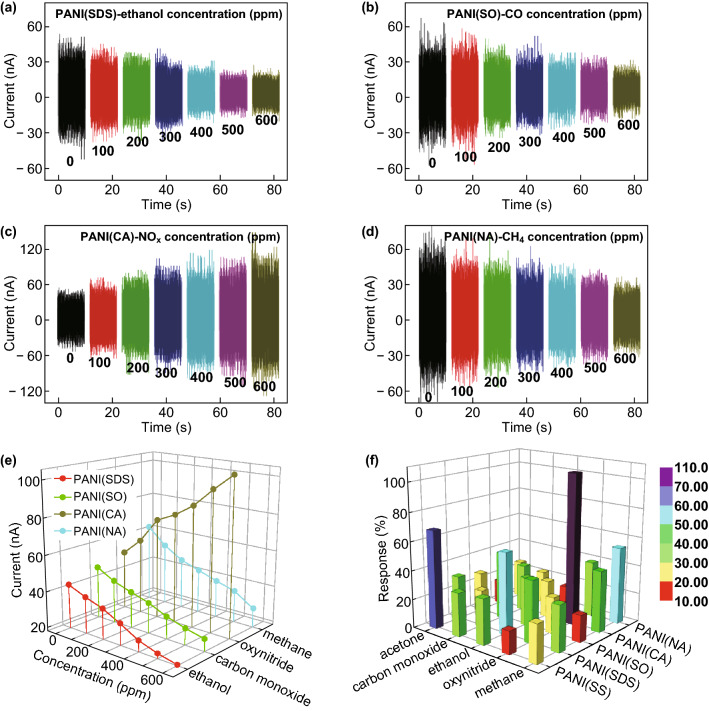
Sensing performances of the five sensing units. The output current of **a** PANI(SDS) unit under different ethanol concentrations. **b** PANI(SO) unit under different CO concentrations. **c** PANI(CA) unit under different NO_*x*_ concentrations. **d** PANI(NA) unit under different CH_4_ concentrations. **e** The relationship of the four units between the output current and special gas marker concentration. **f** The response of the five sensing units to 600 ppm gases

It has been reported that PANI derivatives doped with diverse dopants demonstrate different selectivities toward various gas markers [23]. The sensing performance of sensing units with different PANI derivatives in their particular gas atmospheres is shown in Fig. 4. With a concentration from 0 to 600 ppm of particular gas markers, the output current of PANI(SDS) in response to ethanol (Fig. 4a) is 42.7, 38.4, 35.0, 30.1, 24.2, 20.8, and 18.4 nA; the output current of PANI(SO) in response to CO (Fig. 4b) is 47.1, 42.0, 38.1, 34.9, 30.5, 27.4, and 24.9 nA; the output current of PANI(CA) in response to NO_*x*_ (Fig. 4c) is 51.0, 60.2, 73.9, 78.7, 85.4, 95.7, and 104.6 nA; and the output current of PANI(NA) in response to CH_4_ (Fig. 4d) is 62.0, 52.8, 46.2, 42.7, 38.9, 35.9, and 28.8 nA, respectively. Figure 4e shows the relationship of the four sensing units between the output current and the concentration of gas markers. The response of the five sensing units to 600 ppm of particular gas markers is shown in Fig. 4f, with the gas marker of PANI(SS), PANI(SDS), PANI(SO), PANI(CA), and PANI(NA) as acetone, ethanol, CO, NO_*x*_, and CH_4_, respectively. The maximum response of each sensing unit to 600 ppm of gas marker is 68.2%, 56.9%, 47.1%, 105.1%, and 53.5%, respectively. Similar results are found in several previous studies, which can be attributed to the dopant-induced change of morphology and conformation of PANI derivatives [44, 45]. These results indicate the potential application of the device as a self-powered exhaled breath analyzer for disease diagnostics.

**Fig. 5:**
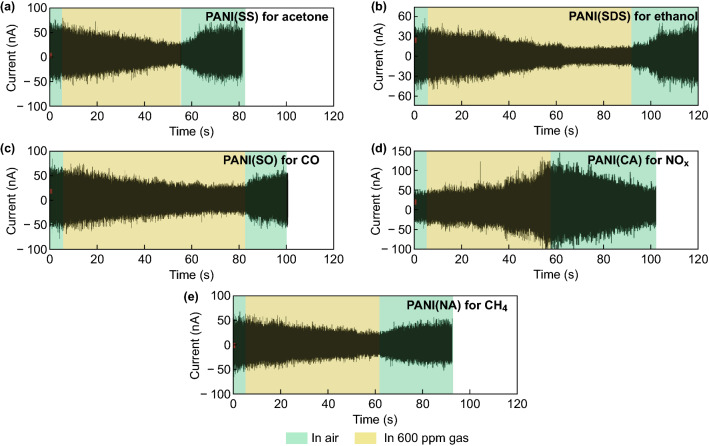
Response/recovery processes of the sensing units. **a** PANI(SS) for acetone. **b** PANI(SDS) for ethanol. **c** PANI(SO) for CO. **d** PANI(CA) for NO_*x*_. **e** PANI(NA) for CH_4_

Figure 5 shows the real-time continuously measured current profiles of the five sensing units to illustrate the dynamic response/recovery process with particular gas markers, all of which exhibit a slow response and a fast recovery time. The response time of PANI(SS), PANI(SDS), PANI(SO), PANI(CA), and PANI(NA) is ~ 50, 63, 67, 54, and 52 s, while the recovery time is ~ 15, 24, 18, 40, and 28 s, respectively. Such relatively slow response/recovery process is common in room-temperature gas sensors [46, 47]. The recovery process is faster than the response time, which may be ascribed to the high-speed gas flow measurement conditions for desorption of gas molecules from PANI film. This proves that the approach is an effective method for real-time exhaled breath detection.

**Fig. 6:**
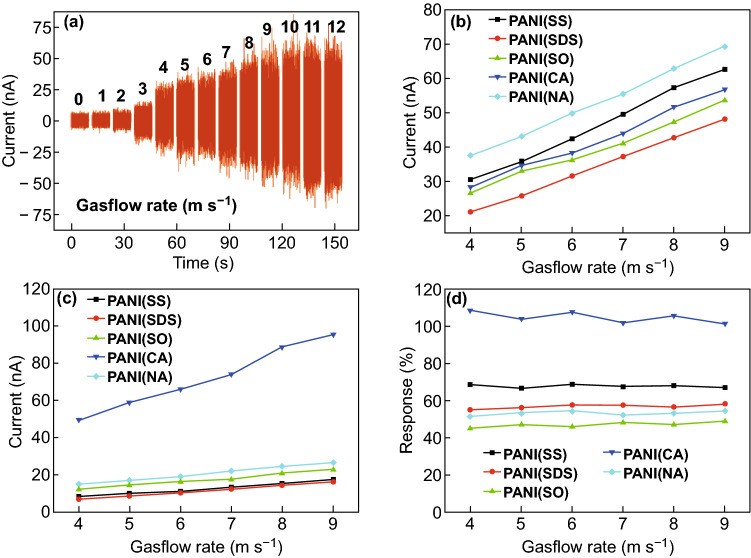
Sensing performances of the five units under different gas flow rates. **a** The output current of PANI(SS) unit under airflow rates in the range of 0–12 m s^−1^. **b** The output current of five sensing units under airflow rates in the range of 4–9 m s^−1^. **c** The output current of five sensing units in response to 600 ppm special gas marker under gas flow rate in the range of 4–9 m s^−1^. **d** The relationship of the five sensing units between responses and gas flow rates

The piezoelectric output current of PANI/PVDF bellows is affected by gas flow rate. Therefore, the influence of the gas flow rate is investigated, and the results are shown in Figs. S4 and 6. Figure S4 shows that the frequency of output current increases under airflow rate in the range of 1–10 m s^−1^. Figure 6a shows the output current of the PANI(SS) unit with increasing airflow rate. The sensing unit outputs a tiny current with airflow rates lower than 3 m s^−1^, while the output current significantly increases with airflow rates over 3 m s^−1^. Under low gas flow rates, vibrations are mainly induced by turbulent buffeting, while under high gas flow rates, they are mainly induced by elastic excitation. The vibrations rapidly increase with tiny increments in the gas flow rate when the gas flow rate is just over a certain threshold [48]. With an airflow rate ranging from 4 to 9 m s^−1^, the output current is 27.6, 33.2, 36.3, 41.0, 47.2, 53.7, and 55.9 nA, respectively, showing a linearly increasing trend. The output current reaches saturation with airflow rates over 9 m s^−1^. Figure 6b, c shows the output current of the five sensing units with air and with 600 ppm of gas markers, respectively, indicating that the output current of every sensing unit linearly increases both in airflow and gas markers with the gas flow rate ranging from 4 to 9 m s^−1^. Interestingly, the calculated responses are almost maintained at a constant level, as shown in Fig. 6d. The result shows that the gas flow rate has a negligible effect on gas response, further supporting the possibility of the device utilization in self-powered exhaled breath analysis.

**Fig. 7:**
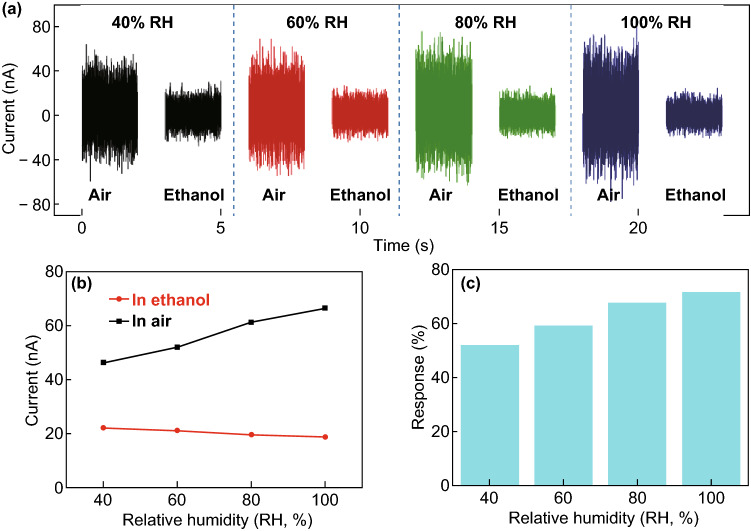
Humidity influence on ethanol response in the PANI(SDS) sensing unit. **a** The output current of the PANI(SDS) sensing unit under airflow and 600 ppm ethanol gas flow in different humidity conditions (40%, 60%, 80%, and 100% RH). **b** The relationship between output current and relative humidity in airflow and 600 ppm ethanol gas flow. **c** The relationship between response and relative humidity

For breath analysis applications, humidity dependence on gas response of the device is very important. Figure 7 shows the influence of humidity on the ethanol response of the PANI(SDS) sensing unit. The relative humidity (RH) was controlled as 40%, 60%, 80%, and 100% RH, respectively. The output current of the PANI(SDS) sensing unit under airflow and 600 ppm ethanol gas flow in different humidity conditions is shown in Fig. 7a. From Fig. 7b, it can be observed that the output current increases with increasing RH in airflow, but decreases with increasing RH in ethanol gas flow. When the device is in humid airflow, water molecules can infiltrate pores in the PANI film to enhance conductivity and increase the output current. When the device is in humid ethanol gas flow, ethanol molecules react with PANI chains to decrease the doping level of PANI and liberate more infiltrated water molecules, leading to a significant increase in the resistance of PANI [49, 50]. As shown in Fig. 7c, the ethanol response increases with increasing humidity, even in high humidity levels. These results reveal that the device has potential application under high humidity levels in gas flow, such as in the case of human breath analysis.

**Fig. 8:**
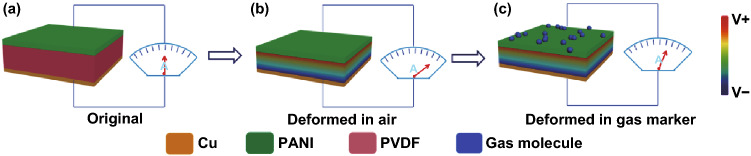
Working principle of the device. **a** The original state of polarized PVDF. **b** Measuring the output current in air atmosphere. **c** Measuring the output current in gas marker atmosphere

The working mechanism of the self-powered exhaled breath analyzer is simply shown in Fig. 8. The schematic diagram of the induced piezoelectric effect of PVDF is shown in Fig. 8a. Under blowing-induced deformation, the polarized dipoles in PVDF become parallel-aligned to create surface charge separation. According to the fundamental piezoelectric theory, the generated open-circuit voltage (*V*_OC_) and short-circuit current (*I*_SC_) of piezoelectric PVDF can be written as [33]:

2 $$ V_{\text{OC}} = g_{33} \sigma Yd; $$

3 $$ I_{\text{SC}} = d_{33} YA\varepsilon , $$

where *g*_33_ is the piezoelectric voltage constant, *d*_33_ is the piezoelectric coefficient, *Y* is Young’s modulus, *σ* is the strain in perpendicular direction, *d* is the thickness, *A* is the effective cross-sectional area, and *ε* is the applied strain. When the device is under gas flow, a current can be measured in the external circuit (Fig. 8b). While the blowing maintains a constant gas flow rate, the generated piezoelectricity of PVDF will be stable, and the resistance of PANI electrodes can be significantly influenced by gas species and concentration in the gas flow [39], giving rise to the variation of measured output current in the external circuit (Fig. 8c). To prove this mechanism, the *I*–*V* curve of the PANI(SDS) sensing unit is measured under 0, 300, and 600 ppm ethanol gas flow, shown in Fig. S5, and demonstrates the same response compared with self-powered gas-sensing performances. The interaction between gas molecules and PANI is multiform: For small molecules (CO and CH_4_), the increased resistance can be attributed to chain expansion induced by interposition of gas molecules into PANI [51–53]; for acetone and ethanol, weak chemical interactions between gas molecules and PANI dominate the decrease in doping level, leading to the increased resistance [54, 55]; and for NO_*x*_, a well-known oxidizing gas, PANI will be oxidized and positively charged by transferring electrons to NO_*x*_ molecules, resulting in the decreased resistance of PANI and increase in output current [56]. When the atmosphere returns to air, adsorbed gas molecules are removed from PANI chains, and thus measured current in the external circuit recovers to its original value. Figure S6 shows the SEM image of PANI(NA) upon exposure to CH_4_, indicating that the surface morphology is significantly influenced by interposed gas molecules. Figure S7 shows the thickness of PANI(NA) before and after exposure to NH_4_, showing that the thickness is slightly affected by gas adsorption as well.

**Fig. 9:**
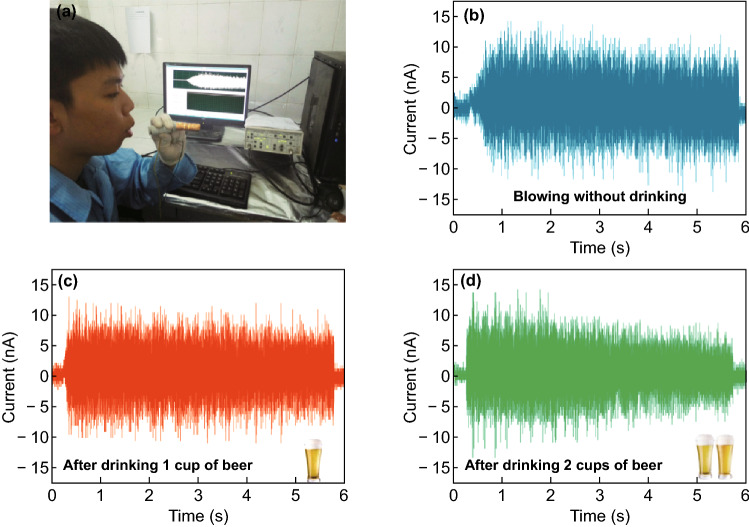
Demonstration of self-powered PANI/PVDF breath analyzer. **a** Photograph of the measurement platform. **b**–**d** The output current of the device in response to blowing by an adult. **b** Without drinking, **c** after drinking one cup of beer, and **d** after drinking two cups of beer

The blowing-driven PANI/PVDF bellows as an exhaled breath analyzer is demonstrated to detect ethanol in exhaled gas, as shown in Fig. 9. As ethanol gas presents itself in the exhaled breath of fatter liver patient, a healthy adult can mimic a fatty liver patient after drinking a certain amount of beer (Fig. 9a). The blowing is sustained for 5 s, and the highest gas flow rate is ~ 3 m s^−1^. The output current of the PANI(SDS) sensing unit is measured to represent ethanol concentration. As the adult blows into the self-powered analyzer prior to drinking beer (Fig. 9b), the output current is 8–12 nA. (The blowing rate is not constant.) As the adult blows into the self-powered analyzer after drinking one and two cups of beer, the output current drops from 10 to 7 nA (Fig. 9c) and from 10 to 5 nA (Fig. 9d), respectively. These results demonstrate that the PANI/PVDF piezo-gas-sensing arrays can be used as breath analyzer for a potential diagnostics application. It is worth noticing that although the sensor arrays can selectively detect several gas markers, the sensitivity and detection limit are insufficient for practical use in the current case. In the future, more work needs to be done to detect ppb-level gas markers, which will involve constructing composites of PANI derivatives and metal oxide nanostructures [57, 58].

## Conclusions

In summary, a blowing-driven bellows based on a PANI/PVDF piezo-gas-sensing array has been presented as noninvasive and self-powered exhaled breath analyzer for potential diagnostic applications. The device works by converting energy from blowing of exhaled breath into electrical sensing signals without any external power sources. Five sensing units in a single device can be used for diagnosis of liver cirrhosis, airway inflammation, diabetes, and asthma by detecting the gas markers in exhaled breath at concentrations in the range from 0 to 600 ppm. The sensing units exhibit excellent room-temperature response/recovery kinetics, and the response is maintained constant under different gas flow rates. The working principle can be attributed to the coupling of in-pipe gas-flow-induced piezoelectric effect of PVDF and gas-sensing properties of PANI electrodes. In addition, the device is demonstrated for detecting ethanol concentration in exhaled breath. This work launches a new working principle in the exhaled breath detection field and greatly advances the applicability of self-powered systems.

## Electronic supplementary material

Below is the link to the electronic supplementary material.

Supplementary material 1 (PDF 559 kb)

Supplementary material 2 (MP4 10182 kb)
