# Supplementary material for: A Self-Powered Breath Analyzer Based on PANI/PVDF Piezo-Gas-Sensing Arrays for Potential Diagnostics Application
Source: Nanomicro Lett. 2018 Nov 12;10(4):76. doi: 10.1007/s40820-018-0228-y (PMC6244558; doi:10.1007/s40820-018-0228-y)
Supplement: Supplementary file 1 — Supplementary material 1 (PDF 559 kb) [file 40820_2018_228_MOESM1_ESM.pdf]

Supporting Information for

A Self-Powered Breath Analyzer Based on PANI/PVDF Piezo-Gas-Sensing Arrays for Potential Diagnostics Application

Yongming Fu[1, 2, 3], Haoxuan He[3], Tianming Zhao[3], Yitong Dai[3], Wuxiao Han[3], Jie Ma[2], Lili Xing[1, 3, *], Yan Zhang[1, *], Xinyu Xue[1, 3, *]

[1]School of Physics, University of Electronic Science and Technology of China, Chengdu 610054, People's Republic of China

[2]College of Physics and Electronics Engineering, Shanxi University, Taiyuan 030006, People's Republic of China

[3]College of Sciences, Northeastern University, Shenyang 110004, People's Republic of China

*Corresponding authors. E-mail: xinglili@mail.neu.edu.cn (L. Xing); zhangyan@uestc.edu.cn (Y. Zhang); xuexinyu@mail.neu.edu.cn (X. Xue)

Supplementary Figures

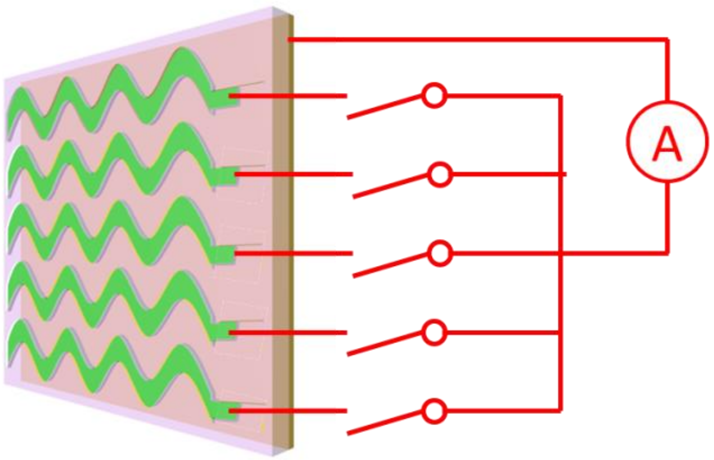

Fig. S1 Schematic diagram of electrode configuration for current measurements

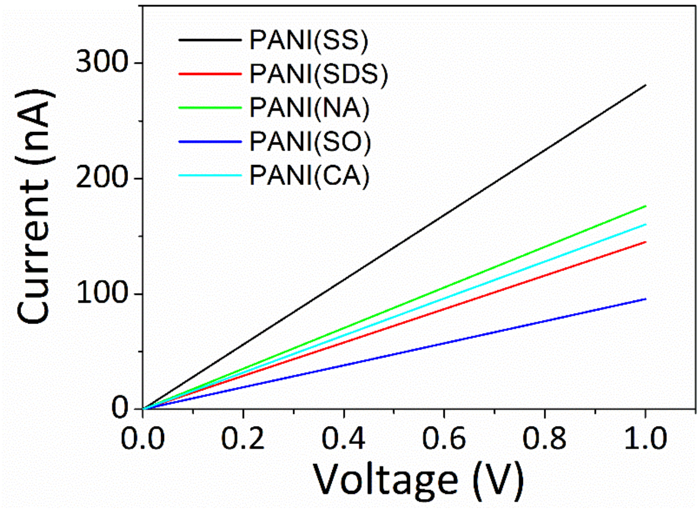

Fig. S2 *I-V* curves of the five PANI derivatives

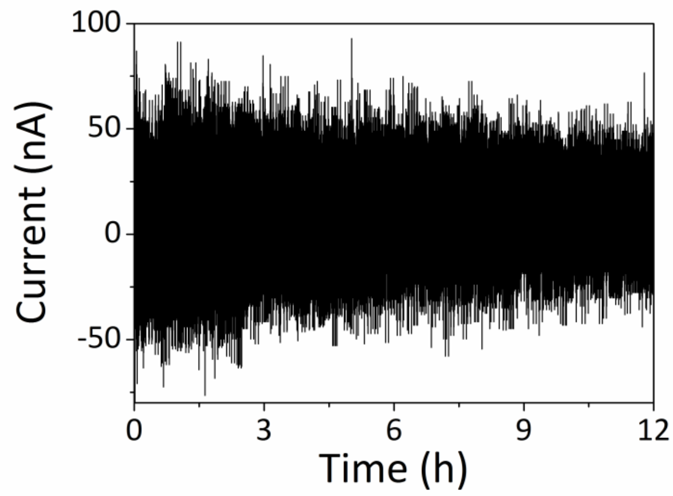

Fig. S3 Long-term stability

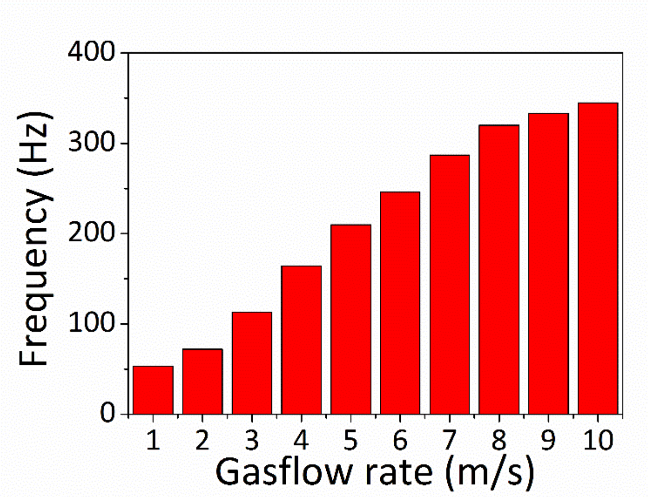

Fig. S4 The relationship between current frequency and gasflow rate

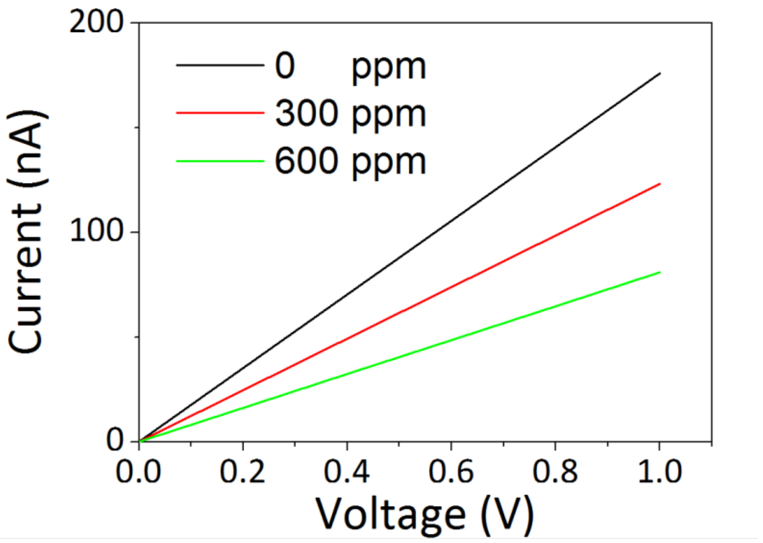

Fig. S5 *I-V* curve of PANI(SDS) sensing unit under 0, 300, and 600 ppm ethanol gasflow

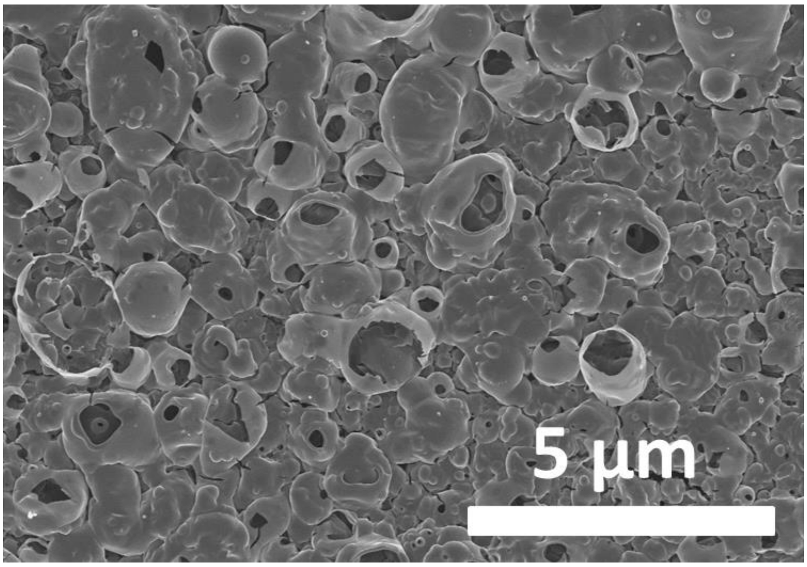

Fig. S6 SEM image of PANI(NA) upon exposure to CH_4

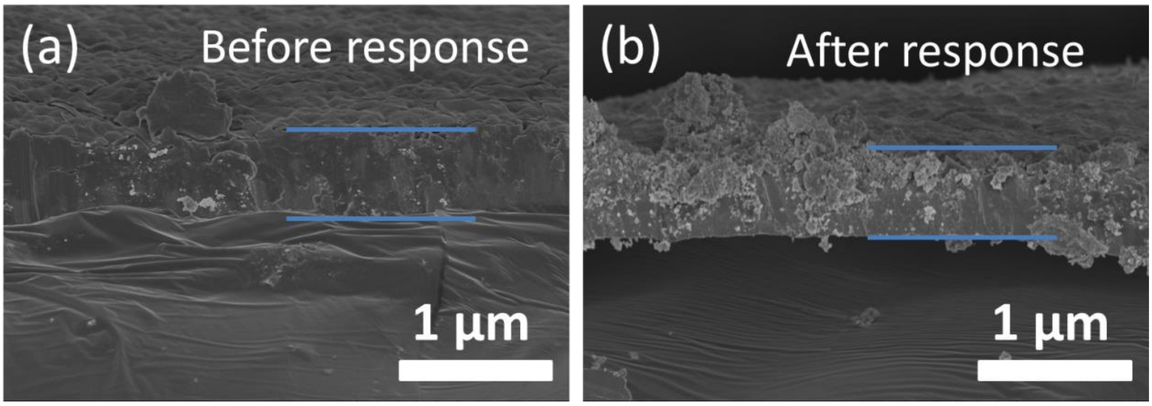

Fig. S7 Cross-view SEM images of PANI(NA) **a** before and **b** after exposed to NH_4
